# Therapeutic Targeting of c-Myc in T-Cell Acute Lymphoblastic Leukemia (T-ALL)

**DOI:** 10.18632/oncotarget.1873

**Published:** 2014-03-27

**Authors:** Marie Loosveld, Rémy Castellano, Stéphanie Gon, Armelle Goubard, Thomas Crouzet, Laurent Pouyet, Thomas Prebet, Norbert Vey, Bertrand Nadel, Yves Collette, Dominique Payet-Bornet

**Affiliations:** ^1^ Centre d'Immunologie de Marseille-Luminy, Aix-Marseille Université UM 2, 13288 Marseille, France; ^2^ INSERM UMR 1104; ^3^ CNRS UMR 7280, 13288 Marseille, France; ^4^ Laboratoire Hématologie, APHM, Marseille, France; ^5^ Centre de Recherche en Cancérologie de Marseille (CRCM) Inserm UMR 1068; Institut Paoli-Calmettes; Aix-Marseille Université UM 105; CNRS UMR 7258, Marseille, France; ^6^ Département d'hématologie, Institut Paoli-Calmettes, Marseille, France

**Keywords:** T-ALL, MYC, JQ1, SAHA

## Abstract

T-ALL patients treated with intensive chemotherapy achieve high rates of remission. However, frequent long-term toxicities and relapses into chemotherapy-refractory tumors constitute major clinical challenges which could be met by targeted therapies. c-MYC is a central oncogene in T-ALL, prompting the exploration of the efficacy of MYC inhibitors such as JQ1 (BET-bromodomain inhibitor), and SAHA (HDAC inhibitor). Using a standardized *ex vivo* drug screening assay, we show here that JQ1 and SAHA show competitive efficiency compared to inhibitors of proteasome, PI3K/AKT/mTOR and NOTCH pathways, and synergize in combination with Vincristine. We also compared for the first time the *in vivo* relevance of such associations in mice xenografted with human primary T-ALLs. Our data indicate that although treatments combining JQ1 or SAHA with chemotherapeutic regimens might represent promising developments in T-ALL, combinations will need to be tailored to specific subgroups of responsive patients, the profiles of which still remain to be precisely defined.

## INTRODUCTION

T-cell acute lymphoblastic leukemias (T-ALL) are aggressive proliferations of transformed T-cell progenitors. Although intensification of chemotherapeutic schedules greatly improved prognosis in the past 10 years, ~30% of cases relapse within the first 2 years following diagnosis [1]; for long-term survivors, acute and lasting toxicities remain important issues underlining the critical need of more adapted/targeted therapies, and better risk stratification. This, however, requires a detailed understanding of T-ALL oncogenic networks, and of escape pathways involved in acquisition of chemoresistance.

Among the numerous oncogenes/tumor suppressors reported in T-ALL, MYC has recently gained a central role [2-5]. MYC alterations are rarely found in T-ALL (<5%), yet indirect upregulation by multiple frequently deregulated pathways makes it one of the most frequently activated oncogenes in T-ALL. In ~50% T-ALLs, increased MYC transcription results from NOTCH mutations [2-4]. In another large fraction of cases, MYC is activated post-translationally, via mutations in FBXW7 or PTEN/PI3K/AKT pathway, impairing sequential ubiquitinations or phosphorylations driving its degradation [6-8]. By providing many escape routes for resistance to therapies targeting afferent pathways (*e.g.* γ-secretase or AKT inhibitors) [8-10], the complexity of MYC circuitry supports the rationale of directly targeting MYC (or direct MYC regulators).

## RESULTS AND DISCUSSION

HDAC inhibitors (*e.g.* SAHA) have been shown to decrease MYC expression levels, although the mechanism is still unclear and undoubtedly non-specific [11]. More recently, the development of BET-bromodomain inhibitors such as JQ1, and the demonstration that it can efficiently inhibit MYC expression via disruption of BRD4-containing transcriptional elongation complexes has triggered great interest [5, 12-15].

**Figure 1:**
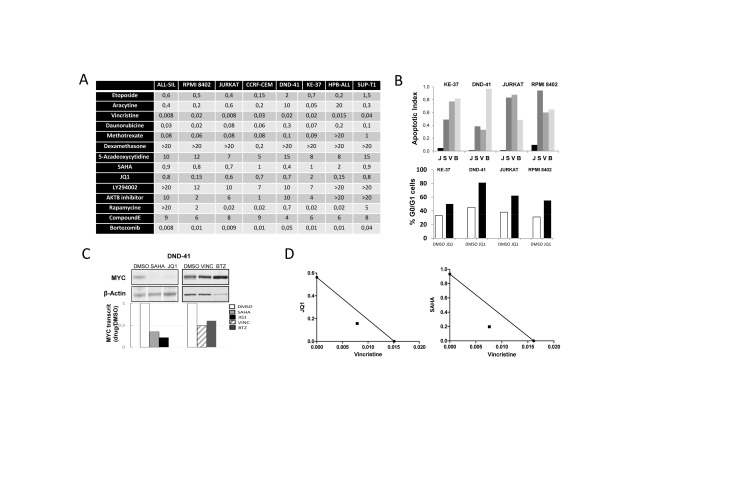
*Ex vivo* evaluation of drug treatments (A) Drug screening. EC50 are reported in µM. (B) T-ALL cell lines were incubated for 48H with 1µM JQ1 (J), 1µM SAHA (S), 50 nM Vincristine (V), 10 nM Bortezomib (B) or the vehicle DMSO, and cellular/molecular analysis were performed. Top: cell apoptosis was monitored by FACS using Annexin V/7-AAD labelling; histograms report the ratio of apoptotic cells treated with drugs versus DMSO. Bottom: cell cycle analysis of T-ALL cell lines treated with JQ1 or DMSO. Cells were labelled with BrdU and 7-AAD, and analyzed by FACS; histograms report the percentage of cells in G0/G1 phase (see Supplemental Fig. S2 for FACS dot plots); (C) Protein extracts and cDNAs were prepared from drug-treated DND41 cells to analyze MYC protein levels by western blot and MYC transcript levels by RQ-PCR. Transcripts are reported as the ratio of MYC transcripts (relative to ABL) from drug-treated cells versus control (for other cell lines see Supplemental Fig. S3). (D) Isobologram representations of the effect of drug combinations Vincristine+JQ1 or Vincristine+SAHA on DND-41 cells viability.

To evaluate the potency of SAHA and JQ1 compared to other inhibitors of key T-ALL pathways, we performed a standardized drug screen of cell proliferation/viability (Supplemental Fig. S1). Eight human T-ALL cell-lines were tested with 8 chemotherapeutic agents and 8 compounds including epigenetic regulators, proteasome inhibitors, PI3K/AKT/mTOR, and NOTCH pathways inhibitors (Supplemental Table S1). Optimal windows of drug molarities were first established by pre-screening, and a more focused panel of 4 serial dilutions was used to determine, for a given T-ALL, the average EC50 value of drugs tested side by side (Fig. 1A). As expected, most non-targeted chemotherapeutic agents displayed efficient inhibitory activity across all cell-lines. Among inhibitors and epigenetic regulators, SAHA and JQ1 combined low EC50, relatively low molarity, and a large spectrum of activity across all T-ALLs (Fig. 1A). *Ex vivo* functional assays on cell cycle and viability showed that while SAHA treatment induced a cytotoxic effect (similar to Vincristine or Bortezomib), JQ1 mainly induced a cytostatic effect (Fig. 1B and Supplemental Fig. S2). This is in line with a previous report on human T-ALL cell-lines [5], but contrasts with *ex vivo* treatments performed on mouse cell-lines and primary human samples (post-culture on stroma and/or *in vivo* amplification in mice), where apoptosis could also be observed upon JQ1 treatment [13]. We next monitored the effect of JQ1 and SAHA on MYC expression. Both transcriptional and protein levels were examined since MYC regulation is impaired at both post-transcriptional and post-translational levels in T-ALL [6]. As expected, relative MYC protein and RNA levels were strongly diminished in all T-ALL cell-lines treated with JQ1 or SAHA, when compared to Vincristine or Bortezomib (Fig. 1C and Supplemental Fig. S3). We next tested whether the inhibitory effect of JQ1 and/or SAHA could be potentialized in combination with Vincristine, frequently included in T-ALL regimens. A synergistic effect was observed for both combinations (Fig. 1D), supporting the rationale of associating BET bromodomain inhibitors or HDAC inhibitors in T-ALL regimens.

**Figure 2:**
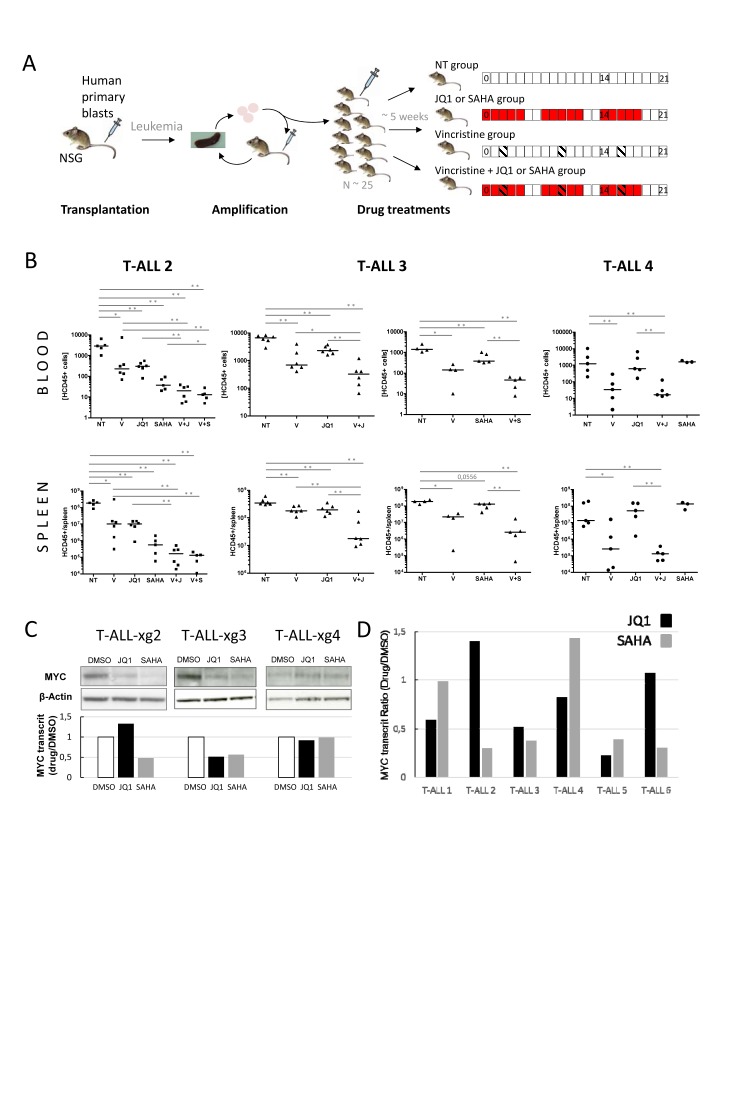
*In vivo* analysis of JQ1/SAHA efficiencies in xenografts of human primary T-ALL (A) Design of xenografts and *in vivo* drug treatment assays. NOD/SCID/γc (NSG) mice are grafted with human primary blasts. Following leukemia development, mice are sacrificed, and blasts from spleens are transplanted in secondary recipient mice for amplification (1-2 rounds). For drug treatments, a set of ~25 NSG mice are engrafted with 1.10^6^ cells issued from the same tumor bulk (from amplification step). Upon appearance of hCD45^+^ human blasts in blood (1-25/µl), mice are divided into pools of 5-6 for drug treatments. Treatments were run over 21 days with indicated schedules: SAHA and JQ1 were injected (ip) 5 days weekly (red boxes); Vincristine was injected (ip) 1 day weekly (hatched boxes); NT: control mice, not treated; Blood samples were drawn at days d0, d14 and d21 post-treatment; mice were sacrificed at d21, and spleens harvested. (B) hCD45^+^ blasts were quantified by FACS; concentrations of hCD45^+^ cells/µl of blood (top plots) and numbers of hCD45^+^ cells per spleen (bottom plots) are shown for T-ALL#2 (■, left plots) T-ALL#3 (▲, middle plots) and T-ALL#4 (●, right plots) xenografted mice. Median values are indicated by horizontal bars. The non-parametric Mann-Whitney test was used to calculate *P* values, differences statistically significant are indicated; * *P* < 0.05, ** *P* < 0.001. V: treated with Vincristine; V+J: treated with Vincristine+JQ1; V+S: treated with Vincristine+SAHA. (C) Western blot analysis of MYC protein expression in xenograft T-ALL#2, T-ALL#3 and T-ALL#4 cells following *ex vivo* treatments by JQ1, SAHA or DMSO; the relative MYC transcript ratio is indicated. (D) Histograms: relative MYC transcript levels of 6 human primary T-ALL cells incubated with 1µM JQ1 (black) or 1µM SAHA (grey). Transcripts are reported as the ratio of MYC transcripts (relative to ABL) from drug-treated cells versus control.

The effect of *in vivo* treatment with these inhibitors has so far not been evaluated in human primary T-ALLs. To further validate the relevance of treatments and associations in an *in vivo* setting, we tested the effect of SAHA, JQ1, Vincristine and combinations in NSG mice xenografted with primary human T-ALL samples (Fig. 2A). Four consecutive fresh samples were directly transplanted into 1-4 mice, without previous amplification or selection on DL1-expressing stroma, to prevent selective bias of NOTCH-addicted (and consequently MYC-dependent) tumors. Among the four xenografted mice, three (T-ALL#2, T-ALL#3 and T-ALL#4) induced T-ALL in less than 4 months. When leukemia-related first symptoms were observed mice were sacrificed and human leukemic cells from spleens harvested for secondary engraftments (amplification step). Then, leukemic blasts of T-ALL#2, T-ALL#3 and T-ALL#4 from secondary xenografts were transplanted (10^6^ cells/mouse) into 25 recipient mice each. Upon appearance of hCD45^+^ blasts in blood (~5 weeks, 1-25 hCD45^+^/µl), pools of 5-6 mice were mock- or drug-treated. For each mouse, circulating hCD45^+^ blasts were quantified by FACS at day 0, and d14/d21 post-treatment. At d21, mice were sacrificed, spleens harvested, weighed and blasts quantified as above (Fig. 2B Supplemental Fig. S4). In all xenografted mice, treatment with Vincristine induced a significant decrease in hCD45^+^ leukemic cell proliferation in spleen and blood at all time-points. For T-ALL#2 and #3, treatments with JQ1 also significantly inhibited tumor progression and spleen size/weight. Similar results were obtained with SAHA in T-ALL#3, while in T-ALL#2 SAHA showed the best efficiency. Remarkably, Vincristine+JQ1 or SAHA combinations further decreased progression, in line with the synergistic effect observed in *ex vivo* assays. By contrast, treatments of T-ALL#4 with JQ1 or SAHA did not induce significant growth inhibition, and Vincristine+JQ1 combination did not enhance the inhibitory effect of Vincristine alone. Importantly, this differential effect of JQ1 or SAHA treatment *in vivo* could not be foreseen on the basis of an *ex vivo* apoptosis assay (Supplemental Fig. S5) where efficiency was uniform, as previously reported [13]. Nonetheless, this differential effect between patients was coherent with MYC expression in transplanted tumors (medium/high in T-ALL#2 and 3, low in T-ALL#4, Fig. 2C). This suggests that at least part of the effect of JQ1 and SAHA on leukemia inhibition *in vivo* is directly or indirectly mediated through MYC or MYC-dependent pathways. This also suggested that contrary to T-ALL cell lines, most of which express high levels of MYC (not shown, potentially as a result of selection/adaptation in *ex vivo* cell culture) and to experimental T-ALL mouse models of Notch activation (in which MYC activation is systematic and homogeneous), primary human T-ALL might display more complex/diverse MYC addiction. In support of this possibility, the effect of JQ1 and SAHA on MYC modulation *ex vivo* appeared heterogeneous in a series of 6 human primary T-ALLs, with 2/6 showing significant MYC decrease with both JQ1 and SAHA (#3,#5), 3/6 showing significant decrease with only one of the 2 drugs (#1,#2,#6) and 1/6 (#4) showing no significant decrease with any of them (Fig. 2D).

Altogether our data indicate that although treatments combining JQ1 or SAHA with chemotherapeutic regimens might represent promising developments in T-ALL, combinations will need to be tailored to specific subgroups of patients, the profiles of which still remain to be precisely defined. Although MYC itself might constitute one element of these profiles, it will undoubtedly not be the sole, considering both the extraordinary complexity of MYC regulatory afferent and effector pathways and the multiple other targets of BET-bromodomain and HDAC inhibitors. Further studies on large cohorts of xenografted primary T-ALL samples (and systematic comparison with *ex vivo* assays) combined with extensive sample characterization will be necessary to identify the key components of such profiles, and ultimately determine whether a sufficiently large subgroup of patients could be eligible and benefit from such combination therapies.

## METHODS

### Human samples and Mice

Peripheral blood or Bone Marrow samples from T-ALLs patients were collected from La Timone Hospital and Paoli-Calmettes Institute (Marseille, France). Informed consent was obtained from patients or relatives in accordance with the Declaration of Helsinki, with institutional review board approval of involved hospitals. Blasts were isolated by Ficoll-Hypaque centrifugation. Mice were bred and maintained in specific-pathogen-free conditions in accordance with institutional guidelines. Detailed methods are provided in Supplemental data.

## SUPPLEMENTARY DATA, TABLE AND FIGURES


