# Supplementary material for: Therapeutic Targeting of c-Myc in T-Cell Acute Lymphoblastic Leukemia (T-ALL)
Source: Oncotarget. 2014 Mar 27;5(10):3168–72. doi: 10.18632/oncotarget.1873 (PMC4102800; doi:10.18632/oncotarget.1873)
Supplement: Supplementary file 1 [file oncotarget-05-3168-s001.pdf]

Supplemental Data

'Therapeutic Targeting of c-Myc in T-Cell Acute Lymphoblastic Leukemia (T-ALL)'
Loosveld et al.

- Supplemental Materiel & methods
- Supplemental references
- Supplemental Table S1 : Compounds used in the drug screening
- Supplemental Figure S1: Drug screening.
- Supplemental Figure S2: Apoptosis and cell cycle analysis of T-ALL cell lines.
- Supplemental Figure S3: Impact of drug treatments on MYC expression.
- Supplemental Figure S4: Drug treatments of NSG mice engrafted with human primary T-ALL.
- Supplemental Figure S5: Apoptosis analysis of human primary T-ALL cells.

Supplemental Materiel and methods

T-ALL cell lines

Human T-ALL cell lines were cultured at 37°C with 5% CO2 in RPMI media supplemented with 10% fetal bovine serum (Lonza) and antibiotics (penicillin 100 U/mL; streptomycin 50 µg/mL).

Drug screening

Drug screening strategy is described in Fig. S1. Typically, drugs (Table S1) are distributed in a 96 well plate and incubated with 1-2.10^5 cells of choice. T-ALL cell lines are incubated at 37°C during 72h. The effect of drugs on cell viability/proliferation is defined by measuring intracellular ATP levels using CellTiter-Glo chemiluminescence kit (Promega) and a Centro XS3 LB 960 plate reader (Berthold technologies), as previously described [1]. Values were normalized to DMSO control. Dose–response curves were generated and effective dose 50 values (EC50) were calculated using nonlinear regression analysis (Graph Pad Prism).

Synergy was assessed using an experimentally determined constant molar ratio of tested drugs. To establish the most effective concentration range for each agent, EC50 values were

determined. Then in subsequent experiments, cells were treated with serial dilutions of each drug individually and with both drugs simultaneously in a fixed molar ratio. The Calcusyn software (Biosoft, Cambridge, UK) was used to determine whether any synergy existed between the agents using the Chou and Talalay method [2] which takes into account both potency (effective dose 50) and the shape of the dose–effect curve. Accordingly, the combination index (CI) was calculated and analyzed as follows: $CI<1$, $CI=1$ and $CI>1$ indicate synergism, additive effect and antagonism, respectively.

Ex-vivo drug treatment

Cell lines were cultured in 6-well plates at 1.10^6 cells/ml and were incubated in the control medium or with 1 µM JQ1, 1 µM SAHA, 10 nM Bortezomib or 50 nM Vincristine. At 24h, cells were harvested for western blot and RQ-PCR and at 48h for viability and cell cycle quantification. Drug treatments of human primary T-ALL were performed as for cell lines and were stopped after 24 H incubation for molecular analysis and for apoptosis assays.

Molecular and cellular analysis

Real-time Quantitative PCR (RQ-PCR). RNA was extracted from T-ALL cells using the column-based system RNAeasy mini kit (Qiagen, Germany) according to the manufacturer’s instructions. Reverse-transcription was performed with High-capacity cDNA reverse transcription kit (Applied Biosystems), and cDNA was analyzed by real-time PCR (RQ-PCR) on an ABI-PRISM 7500 Fast Real-Time PCR system (Applied Biosystems, CA, USA). PCR reactions were performed in 25 µl of diluted cDNA (10X dilution), 0.3 µmol of each primer and 12.5 µl of SYBR Green Master Mix (Roche). Primer sequences for MYC and ABL cDNA are the following: Q-MYC1A , d(GCAGCGACTCTGAGGAGGAA) ; Q-MYC1B d(CCAGGAGCCTGCCTCTTTT) ; Q-ABL-1A d(TTGTGGCCAGTGGAGATAACACT) and Q-

ABL-1B, d(CTTGGCCATTTTTGGTTTGG). All RQ-PCR were performed in duplicate. To allow comparison between samples, transcript quantification was performed after normalization with ABL from previously generated standard curves using the ∆Ct method and calculated according to the following formula $2\Delta^{(CtABL-Ctgene)}$.

Immunoblotting analysis. Cells were lysed for 30 min at 4°C in lysis buffer (urea/thiourea buffer: 7M urea, 2M thiourea, 4% CHAPS and 25 mM Tris(hydroxymethyl)anminomethane pH=8.5), and centrifuged at 75000g for 10 min at 4°C. Protein concentrations of supernatants were determined using Bio-Rad protein assay (Bio-Rad). Equivalent protein extract (~60µg) for each sample was separated by SDS-PAGE and transferred to nitrocellulose membrane using Iblot Gel Transfer stacks and Iblot system (Invitrogen). Membranes were blocked in TTBS (137mM NaCl, 2mM KCl, 25mM Tris(hydroxymethyl)aminomethane and 0.1% tween 20) supplemented with 5% non-fat milk and incubated with primary antibodies against MYC (clone 9E10, Santa Cruz Biotechnology Inc.) or Actin (clone I-19, Santa Cruz Biotechnology Inc.) overnight at 4°C with agitation. The secondary Anti-rabbit, -mouse or - goat antibodies (Santa Cruz Biotechnology) conjugated to HRP were added for 1 hour at room temperature. Immunoblots were revealed using ECL western blotting detection reagents (GE Healthcare) and stripped using Restore western blot stripping buffer (Pierce).

Cell apoptosis and cell cycle. Following treatment, cells were transferred in 96-well plates, washed once with PBS and stained with 100 µL of Annexin V-APC/7-AAD staining solution according to the manufacturer's protocol (BD Pharmingen, APC Annexin V). After 15 min incubation at RT (25°C) in the dark, cells were analyzed by using FACS Canto flow cytometer. The percentage of viable cells (low Annexin V-APC/low 7-AAD), early apoptotic cells (high Annexin V-APC/low 7AAD), and late apoptotic/necrotic cells (high annexin V-

APC/high 7 AAD) was determined. BrdU incorporation assays were performed according to the manufacturer's protocol (BD Pharmingen, APC-BrdU flow kit) with cells pulsed with BrdU for 20 min. Cells were co-stained with 7-AAD for DNA content measurement.

Xenograft and in vivo drug treatment

Mice were bred and maintained in specific-pathogen-free conditions in CIML or CRCM animal facilities in accordance with institutional guidelines. Human primary T-ALL blasts (1.10^6 cells per mouse) were transplanted by intravenous injections in healthy NSG (NOD.Cg-*Prkdc* ^{scid} *II2g* ^{tm1Wji} /SzJ) mice (Charles River, UK). T-ALL onset was monitored by retro-orbital blood sampling and FACS analysis. Typically 100µl of blood were first incubated with red cell lysis buffer (StemCell Technologies) and then incubated with anti-human CD45-APC-Cy7 (BD Pharmingen, ref. 557833) or Pacific blue anti-human CD45 (Biolegend, ref. 304029) and anti-murine CD45-PE (BD Pharmingen, ref. 553081) or anti-murine CD45-APC-efluor780 (ebiosciences ref. 47-0451-82) antibodies, after immunolabelling cells were resuspended in 100 µl FACS buffer (PBSX1, 5% FCS) and 10 µl of counting beads (CountBright absolute, Invitrogen) were added for absolute hCD45+ cell counts. Once T-ALL arose and/or when leukemia-related first symptoms (hunchbacked, significant weight loss or ruffled coat) were observed, mice were sacrificed and human leukemic cells from the spleen were engrafted in secondary recipient NSG mice. Drug treatments were performed using secondary or tertiary transplanted mice. JQ1 (Dana-Farber) dissolved in DMSO/10% hydroxypropyl beta cyclodextrin (SIGMA) was injected (50 mg/kg) five days per week. 20 µl of SAHA (100 mg/ml DMSO) were injected per mice 5 days/week. Vincristine (0,25 mg/kg) was administrated once a week. All drugs were administrated by intraperitoneal injections (ip) and no significant weight loss was noticed in any group of mice during the treatment period.

Supplemental references

1. Salmi-Smail C, Fabre A, Dequiedt F, Restouin A, Castellano R, Garbit S, Roche P, Morelli X, Brunel JM and Collette Y. Modified cap group suberoylanilide hydroxamic acid histone deacetylase inhibitor derivatives reveal improved selective antileukemic activity. J Med Chem. 2010; 53(8):3038-3047.

2. Chou TC and Talalay P. Quantitative analysis of dose-effect relationships: the combined effects of multiple drugs or enzyme inhibitors. Adv Enzyme Regul. 1984; 22:27-55.

3. Pui CH and Jeha S. New therapeutic strategies for the treatment of acute lymphoblastic leukaemia. Nat Rev Drug Discov. 2007; 6(2):149-165.

4. Delmore JE, Issa GC, Lemieux ME, Rahl PB, Shi J, Jacobs HM, Kastritis E, Gilpatrick T, Paranal RM, Qi J, Chesi M, Schinzel AC, McKeown MR, Heffernan TP, Vakoc CR, Bergsagel PL, et al. BET bromodomain inhibition as a therapeutic strategy to target c-Myc. Cell. 2011; 146(6):904-917.

5. Filippakopoulos P, Qi J, Picaud S, Shen Y, Smith WB, Fedorov O, Morse EM, Keates T, Hickman TT, Felletar I, Philpott M, Munro S, McKeown MR, Wang Y, Christie AL, West N, et al. Selective inhibition of BET bromodomains. Nature. 2010; 468(7327):1067-1073.

6. Uddin S, Hussain A, Al-Hussein K, Platanias LC and Bhatia KG. Inhibition of phosphatidylinositol 3'-kinase induces preferentially killing of PTEN-null T leukemias through AKT pathway. Biochem Biophys Res Commun. 2004; 320(3):932-938.

7. Chan SM, Weng AP, Tibshirani R, Aster JC and Utz PJ. Notch signals positively regulate activity of the mTOR pathway in T-cell acute lymphoblastic leukemia. Blood. 2007; 110(1):278-286.

8. Cullion K, Draheim KM, Hermance N, Tammam J, Sharma VM, Ware C, Nikov G, Krishnamoorthy V, Majumder PK and Kelliher MA. Targeting the Notch1 and mTOR pathways in a mouse T-ALL model. Blood. 2009.

9. De Keersmaecker K, Lahortiga I, Mentens N, Folens C, Van Neste L, Bekaert S, Vandenberghe P, Odero MD, Marynen P and Cools J. In vitro validation of gamma-secretase inhibitors alone or in combination with other anti-cancer drugs for the treatment of T-cell acute lymphoblastic leukemia. Haematologica. 2008; 93(4):533-542.

10. Huang C, Hu X, Wang L, Lu S, Cheng H, Song X, Wang J and Yang J. Bortezomib suppresses the growth of leukemia cells with Notch1 overexpression in vivo and in vitro. Cancer Chemother Pharmacol. 2012; 70(6):801-809.

Supplemental Table and figures

Name	Function	References
**Etoposide**	Chemotherapy, Topoisomerase II inhibitor	MYLAN[3]
**Aracytine**	Chemotherapy, Antimetabolite	PFIZER[3]
**Vincristine**	Chemotherapy, Antimicrotubule agents	HOSPIRA/TEVA[3]
**Daunorubicin**	Chemotherapy, Topoisomerase II inhibitor	SIGMA-ALDRICH[3]
**Methotrexate**	Chemotherapy, Antimetabolite	TEVA[3]
**Dexamethasone**	Corticosteroids	SIGMA-ALDRICH[3]
**5 Azadeoxycytidine**	Epigenetic modifier, DNA demethylation	SIGMA-ALDRICH[3]
**SAHA**	Epigenetic modifier, Histone deacetylase inhibitor	Home Made[1]
**JQ1**	BET bromodomains inhibitor	DANA-FARBER Cancer Institute[4, 5]
**LY294002**	PI3K inhibitor	CALBIOCHEM[6]
**AKT8 inhibitor**	AKT inhibitor	CALBIOCHEM
**Rapamycine**	mTOR inhibitor	SIGMA-ALDRICH[7, 8]
**CompoundE**	GSI inhibitor	TEBU-BIO[9]
**Bortezomib**	Proteasome inhibitor	LcLABS[10]

Supplemental Table S1 : Compounds used in the drug screening

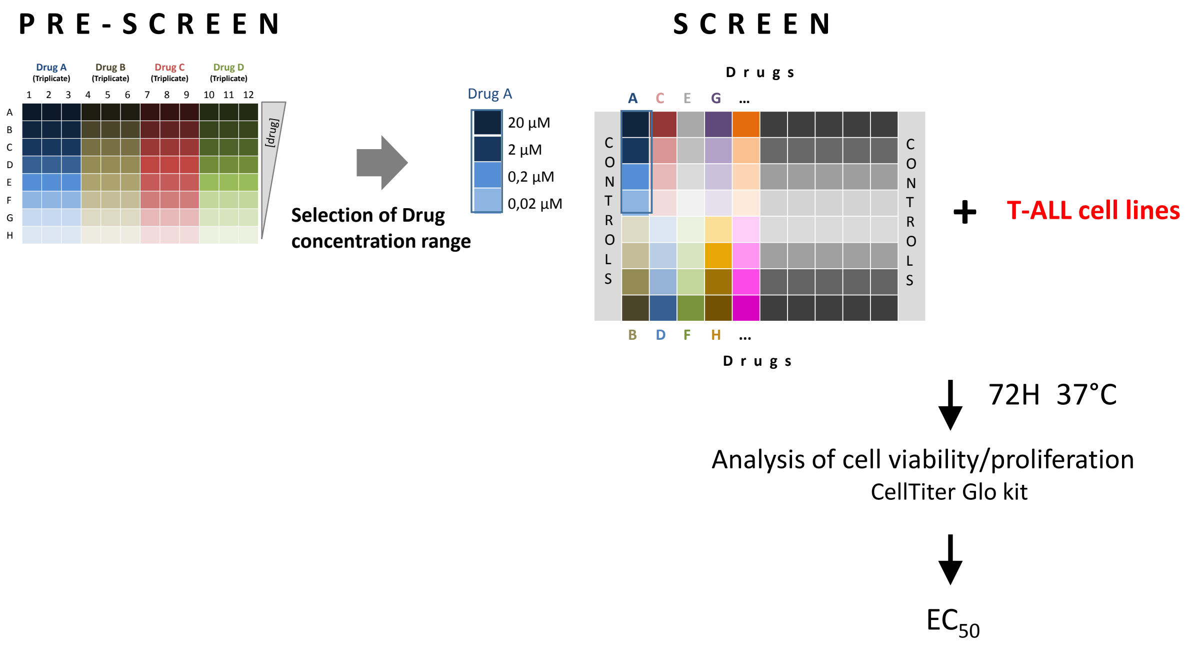

Supplemental Figure S1: Drug screening.

Drugs were first distributed in a 96 wells plate in triplicate at 8 concentrations ranging from 100 µM to 10^{-5} µM (except for Daunorubicin for which a range from 10 µM to 10^{-6} µM was used), then DND-41 cells (1.10^5/well) were added and plates were incubated at 37°C. After 72H, metabolic activity (reflecting cell growth and viability) was monitored by measuring intracellular ATP levels using CellTiter-Glo chemiluminescence reagent. This pre-screen was performed to define a narrow range of drug concentrations that will be used for the screen. Typically for the screen, drugs of interest are each used at 4 concentrations: 20 nM to 20 µM for most drugs; 2 nM to 2 µM for Daunorubicin and Vincristine; 0.2 nM to 0.2 µM for Bortezomib. Then, similarly to the pre-screen, drugs were distributed in a 96 wells plate and were incubated for 72H with ~1.10^5 cells of choice. Finally, samples were analyzed using CellTiter-Glo kit and the concentration for which 50% of the cells were affected (EC_{50}) was determined for each drugs. In the control wells, cells were incubated with culture media supplemented with 5% DMSO.

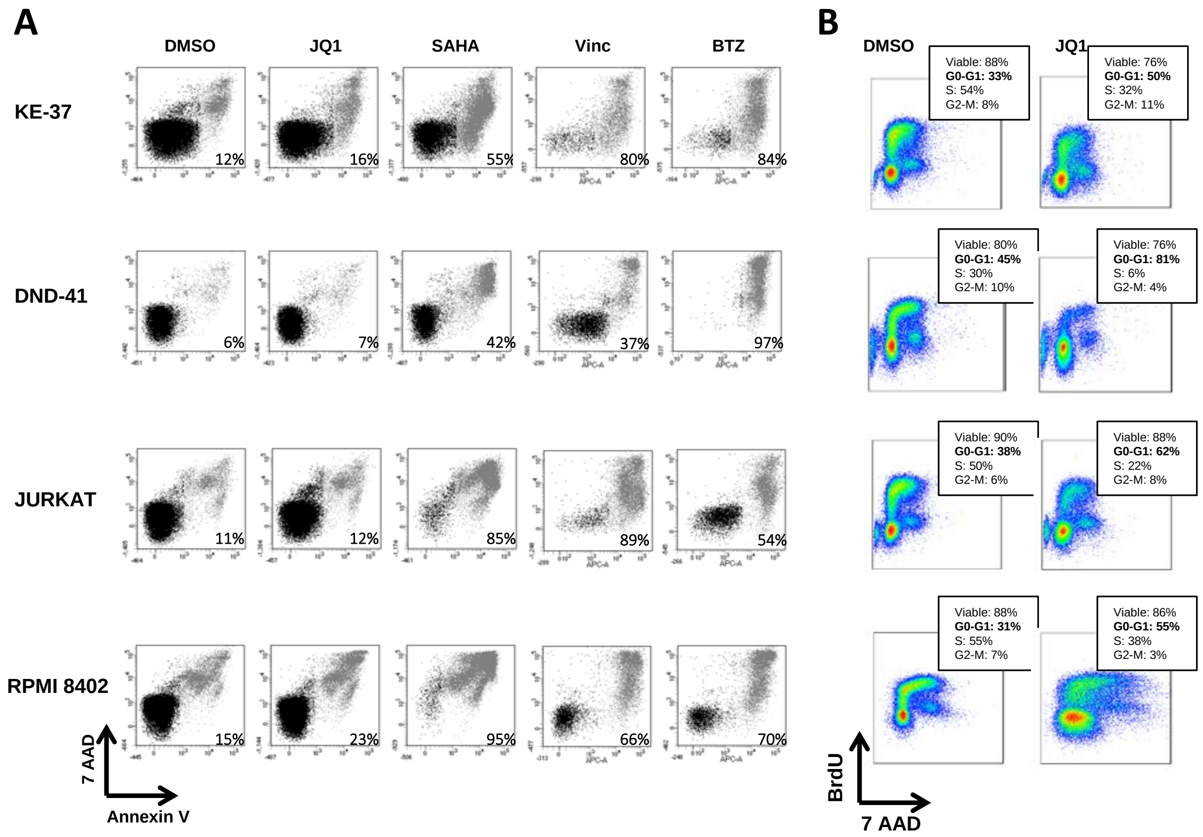

Supplemental Figure S2: Apoptosis and cell cycle analysis of T-ALL cell lines.

FACS dot plots corresponding to histograms shown in Fig 1B.

(A) T-ALL cell lines were labelled with Annexin V/7-AAD. Percentages of annexin V+ cells (grey dots) are indicated. (B) T-ALL cells were labelled with BrdU and 7-AAD; percentages of cells in the various cell cycle phases are indicated.

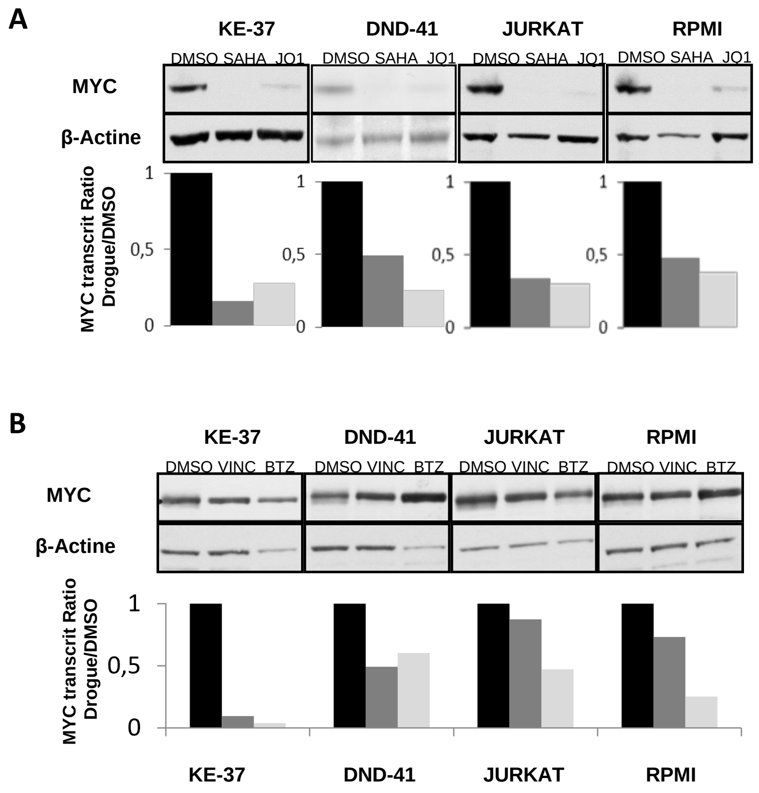

Supplemental Figure S3: Impact of drug treatments on MYC expression.

Analysis of MYC expression as described in Fig. 1C. **(A)** T-ALL cell lines were treated with JQ1 or SAHA. **(B)** T-ALL cell lines were treated with Vincristine (Vinc) or Bortezomib (BTZ). **(A & B)** Top: MYC Western blot. Bottom: histograms of relative MYC transcriptional expression.

A

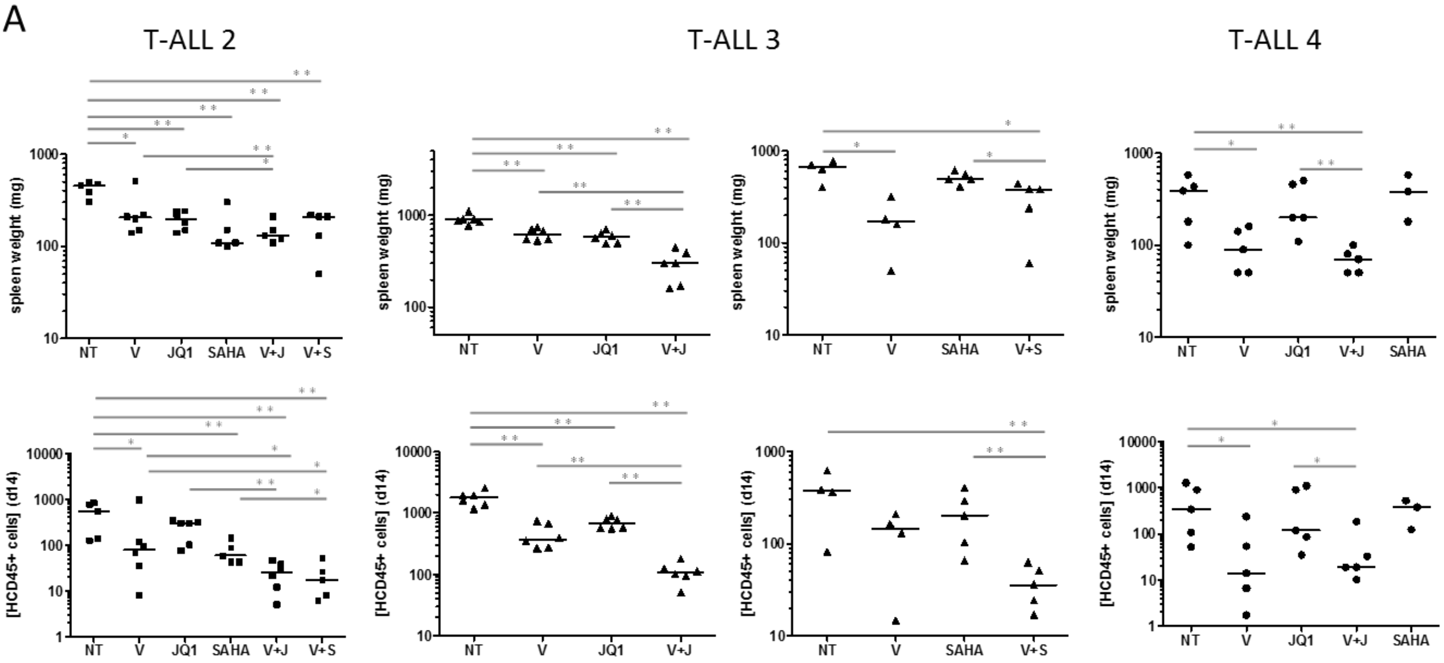

B

		Mean spleen weight
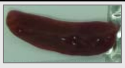	NT	336 mg
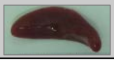	JQ1	294 mg
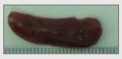	SAHA	380 mg
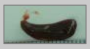	Vinc	98 mg
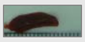	V+J	70 mg
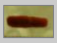	NSG	50 mg

1 cm

Supplemental Figure S4: Drug treatments of NSG mice engrafted with human primary T-ALL.

Supplemental results of assays described in Fig. 2. Drug treatments of mice engrafted with T-ALL #2, #3 and #4. **(A)** Top: plots of spleen weight at d21. Bottom: concentrations of HCD45+ cells/µl of blood at d14. **(B)** Photographs of typical spleens of mice engrafted with T-ALL #4 and treated with drugs reported in the middle column. For each type of treatment the mean spleens weight is indicated.

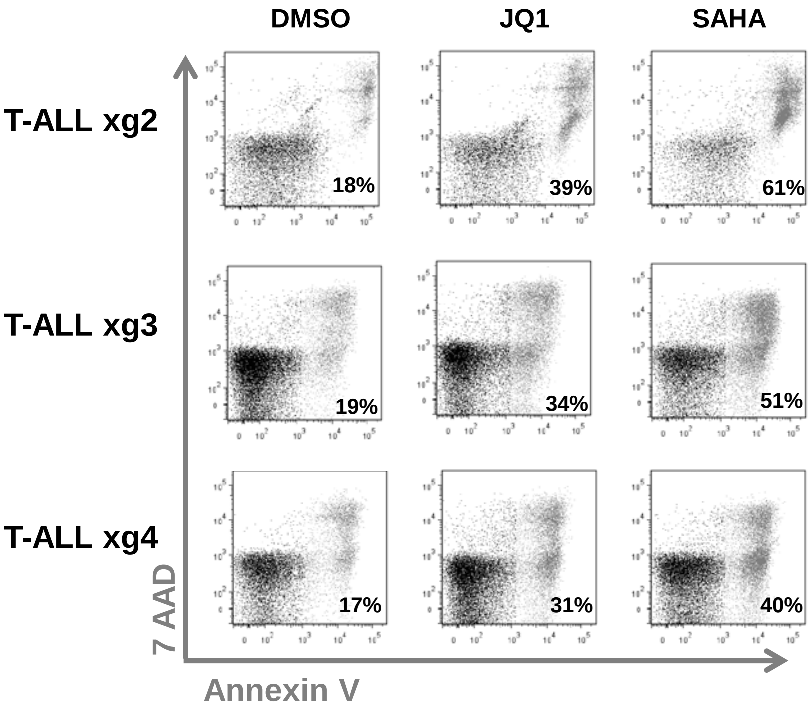

Supplemental Figure S5: Apoptosis analysis of human primary T-ALL cells.

Post-xenograft T-ALL cells (from patients #2, #3 and #4) treated ex-vivo with DMSO, JQ1 or SAHA were labelled with Annexin V/7-AAD and analyzed by FACS. Percentages of annexin V+ cells (grey dots) are indicated.
